# Bacterial Diversity Associated With the Rhizosphere and Endosphere of Two Halophytes: *Glaux maritima* and *Salicornia europaea*

**DOI:** 10.3389/fmicb.2018.02878

**Published:** 2018-11-28

**Authors:** Kosuke Yamamoto, Yuh Shiwa, Taichiro Ishige, Hikaru Sakamoto, Keisuke Tanaka, Masataka Uchino, Naoto Tanaka, Suguru Oguri, Hiromasa Saitoh, Seiya Tsushima

**Affiliations:** ^1^Department of Molecular Microbiology, Faculty of Life Sciences, Tokyo University of Agriculture, Tokyo, Japan; ^2^NODAI Genome Research Center, Tokyo University of Agriculture, Tokyo, Japan; ^3^Department of Northern Biosphere Agriculture, Faculty of Bioindustry, Tokyo University of Agriculture, Hokkaido, Japan

**Keywords:** bacterial diversity, *Glaux maritima*, *Salicornia europaea*, halophyte, endophytic bacteria, rhizosphere bacteria, 16S rRNA, metagenome

## Abstract

Root-associated microbial communities are very important in the adaptation of halophytes to coastal environments. However, little has been reported on microbial community structures related to halophytes, or on comparisons of their compositions among halophytic plant species. Here, we studied the diversity and community structure of both rhizosphere and root endosphere bacteria in two halophytic plants: *Glaux maritima* and *Salicornia europaea*. We sampled the rhizosphere, the root endosphere, and bulk control soil samples, and performed bacterial 16S rRNA sequencing using the Illumina MiSeq platform to characterize the bacterial community diversities in the rhizosphere and root endosphere of both halophytes. Among the *G*. *maritima* samples, the richness and diversity of bacteria in the rhizosphere were higher than those in the root endosphere but were lower than those of the bulk soil. In contrast for *S*. *europaea*, the bulk soil, the rhizosphere, and the root endosphere all had similar bacterial richness and diversity. The number of unique operational taxonomic units within the root endosphere, the rhizosphere, and the bulk soil were 181, 366, and 924 in *G*. *maritima* and 126, 416, and 596 in *S*. *europaea*, respectively, implying habitat-specific patterns for each halophyte. In total, 35 phyla and 566 genera were identified. The dominant phyla across all samples were *Proteobacteria* and *Bacteroidetes*. *Actinobacteria* was extremely abundant in the root endosphere from *G*. *maritima*. Beneficial bacterial genera were enriched in the root endosphere and rhizosphere in both halophytes. *Rhizobium*, *Actinoplanes*, and *Marinomonas* were highly abundant in *G*. *maritima*, whereas *Sulfurimonas* and *Coleofasciculus* were highly abundant in *S*. *europaea.* A principal coordinate analysis demonstrated significant differences in the microbiota composition associated with the plant species and type of sample. These results strongly indicate that there are clear differences in bacterial community structure and diversity between *G*. *maritima* and *S*. *europaea*. This is the first report to characterize the root microbiome of *G*. *maritima*, and to compare the diversity and community structure of rhizosphere and root endosphere bacteria between *G*. *maritima* and *S*. *europaea*.

## Introduction

Salinity affects more than 800 million hectares of the total agricultural land, leading to a decrease of approximately 1–2% of the global arid and semi-arid zones every year (Kouam and Marie Solange Mandou, 2017; Etesami and Beattie, 2018). Many agricultural crops are susceptible to salt stress (Glenn et al., 1991). Future agricultural production in salt-damaged fields, therefore, requires the development of salt-tolerant crops (Etesami and Beattie, 2018). To generate crops that are able to grow on salt-damaged fields, a large amount of basic research has focused on funding and characterizing salt-resistant-related genes in model plants, and using these to improve plant salt tolerance through genetic modification and editing. However, despite numerous studies, only minor success has been achieved, as these approaches have often overlooked the important role of plant–microbe interactions in response to salt stress conditions (Coleman-Derr and Tringe, 2014; Yuan et al., 2016). It is well-known that the plant-associated microbial community plays an important role in adapting plants to extreme environments (Redman et al., 2002; Yuan et al., 2016).

A large number of reports have shown that halotolerant plant growth-promoting rhizobacteria (PGPRs) isolated from halophytes enhance salt tolerance in their host plants. For example, the salt tolerance of *Arthrocnemum macrostachyum* is improved by its endophytic bacteria (Navarro-Torre et al., 2017; Tian and Zhang, 2017). As another example, in *Salicornia strobilacea*, rhizospheric bacteria which can stably colonize the rhizoplane are capable of improving plant growth (Marasco et al., 2016). Furthermore, several halotolerant PGPRs have also been shown to improve the growth of various agricultural crops under salt stress conditions (Etesami and Beattie, 2018). *Micrococcus yunnanensis*, *Planococcus rifietoensis*, and *Variovorax paradoxus*, which are found in halophytes, have been shown to significantly improve salt-stress tolerance in sugar beet (Zhou et al., 2017; Etesami and Maheshwari, 2018). *Pseudomonas* spp. from *Suaeda salsa* have also been shown to be responsible for increasing salt stress tolerance and plant growth in cucumber and rice plants (Yuan et al., 2016). However, further research on the diversity of bacterial communities present in the rhizosphere and the root endosphere of various halophyte species is required before these PGPRs can be used in saline soil-based agriculture (Etesami and Beattie, 2018). Of particular importance is the fact that the diversity of halotolerant PGPRs present in soil with high salt content, as well as the diversity of halophyte-associated endophytic bacteria, depend on both the soils chemical and physical properties, as well as the plant species (Qin et al., 2016; Szymańska et al., 2016). Therefore, we have studied the diversity and community structure of bacteria present in the rhizosphere and the root endosphere of two halophytic plants: *Glaux maritima* (Primulaceae) and *Salicornia europaea* (Chenopodiaceae), which have different degrees of salt tolerance. *G*. *maritima* grows in coastal salt marshes and coastal meadows which are periodically flooded by the sea (Freipica and Ievinsh, 2010). The growth and development of *G*. *maritima* explants is stimulated by 100 mM NaCl and decreased when the NaCl concentration is raised above 200 mM. *S*. *europaea* is one of the highest salt accumulating halophytes and is found in both coastal and inland saline sites. The growth of *S*. *europaea* is significantly enhanced under 3% NaCl conditions and is suppressed in the presence of 5% (856 mM) NaCl, but the explants are able to survive (Yamamoto et al., 2009; Zhao et al., 2016).

In this report, we describe the first study to characterize the rhizosphere and root endosphere bacteria related to *G*. *maritima* and to compare its diversity and community structure with those of *S*. *europaea*. Our results bring new insight into the complex bacteria community in coastal halophytes.

## Materials and Methods

### Sample Collection

Naturally growing *G*. *maritima* were collected from Lake Notoro in the eastern part of Hokkaido, Japan, (44°2′50″N/144°11′25″E) and *S*. *europaea* was collected in a nearby location (44°2′51″N/144°11′20″E) in July 2017 (Supplementary Figure S1). Sampling was conducted under permission from the Hokkaido Prefecture. Sampling was performed according to the method described by Schlaeppi et al. (2014). We excavated whole plants including the surrounding soil in blocks (∼20 cm in length, ∼20 cm wide, and 10–20 cm in depth). The plants in their soil cores were brought to the laboratory, and the root systems were sampled within 12 h of removing the plants from their natural habitat. A total of 20 individual samples representing quadruplicate samples of five plant species were obtained in total, and from these, the rhizosphere and root compartments were separated and used for bacterial community profiling.

### Sample Preparation

Fractionation of the rhizosphere and the root endosphere was performed according to the methods described previously (Schlaeppi et al., 2014; D’Amico et al., 2018). Roots were collected and cut into 3 cm long segments starting 0.5 cm below the root base. The collected roots were placed into 15 mL sterile tubes containing 10 mL PBS-S buffer (130 mM NaCl, 7 mM Na_2_HPO_4_, 3 mM NaH_2_PO_4_, pH 7.0, 0.02% Silwet L-77), and then washed with shaking at 180 rpm for 20 min. The roots were then transferred to a new 15 mL sterile tube, and the soil suspension was centrifuged for 20 min at 4,000 ×*g*. The pellet generated was defined as the rhizosphere (soil-root interface), and frozen in liquid nitrogen for storage at -80°C. After subsequent washing using the same procedure as described above, the roots were transferred to a new 15 mL sterile tube with 10 mL PBS-S buffer and sonicated for 10 min with a water bath sonicator at 40 kHz (Model 5510, Branson Ultrasonics Corporation, Danbury, CT, United States) to enrich for endophytic bacteria present in the roots. The roots were washed in a fresh volume of 10 mL PBS-S buffer using the same procedure described above and then dried on 50 mm diameter Whatman filter paper (GE Healthcare, Pittsburgh, PA, United States), transferred to a new 15 mL sterile tube, and then frozen in liquid nitrogen for storage at -80°C. This root sample, which is enriched in root endophytic bacteria, was defined as the root endosphere (Re) as described by D’Amico et al. (2018). After all plants were harvested from the soil block, the bulk soil samples were collected from 0.5 to 3.5 cm from the soil surface corresponding to a 3 cm root length, frozen in liquid nitrogen, and stored at -80°C. The collected rhizosphere (Rh), root endosphere (Re), and bulk control soil (Bl) samples were used for DNA extraction.

### DNA Extraction, PCR Amplification, and Gene Clone Library Construction

Samples (0.5 g) of each of the Rh and Bl samples were used for DNA extraction. Re samples were frozen in liquid nitrogen and ground to a fine powder with a sterilized mortar and pestle. Following this, 0.5 g of the Re sample was used for DNA extraction. Total DNA was extracted using a NucleoSpin Soil Kit (Macherey-Nagel, Düren, Germany) containing buffer SL1 and enhancer SX, which have been previously used to extract both a high quality and quantity of DNA from paddy soil (Knauth et al., 2013).

The hypervariable V3-V4 regions of the bacterial 16S rRNA gene were amplified using the following primer pairs: forward, 5′-TCGTCGGCAGCGTCAGATGTGTATAAGAGACAGCCTACG GGNGGCWGCAG-3′ and reverse, 5′-GTCTCGTGGGCTCGG AGATGTGTATAAGAGACAG GGACTACHVGGGTWTCTAA T-3′, according to previous methods (Tian et al., 2015; Wei et al., 2017). The nucleotide sequences of the Illumina adapter overhang are shown as the underlined regions, whereas the gene-specific sequences targeting the V3-V4 regions of the prokaryotic 16S rRNA genes are not underlined (Chan et al., 2015). A PCR amplicon library was generated following the Illumina 16S sample preparation guide (16S Sample Preparation Guide, 15044223; Illumina. San Diego, CA, United States). The library quality was assessed on an Agilent 2200 Tapestation (Agilent Technologies, Inc.). The libraries were sequenced as paired-end, 300 bp reads on an Illumina MiSeq benchtop sequencer. All sequence data obtained in this study have been deposited in the DDBJ Sequence Read Archive (DRA) database under accession number: DRA006852.

### Sequence Processing and Analysis

Sequence processing was performed according to the method described by Zabat et al. (2018). Raw paired-end FASTQ files were quality filtered, trimmed, de-noised, and merged using DADA2 (Callahan et al., 2016) in QIIME2 (ver. 2017.11)^1^. Chimeric sequences were identified and removed using the consensus method in DADA2. Using DADA2, sequences were clustered into operational taxonomic units (OTUs) at 100% identity. Taxonomic analysis of OTUs was performed using the QIIME2 q2-feature-classifier plugin with a pre-trained Naïve Bayes classifier on the SILVA 99% OTU database (version 128)^2^ trimmed to the V3-V4 region of the 16S rRNA gene (DeSantis et al., 2006). Multiple sequence alignment and phylogenetic reconstruction were carried out using MAFFT and FastTree, respectively (Price et al., 2009; Katoh and Standley, 2013).

### Statistical Analysis

Subsequent analyses were performed in R ver. 3.4.3 using the phyloseq (McMurdie and Holmes, 2013), pheatmap (Kolde, 2015), and VennDiagram (Chen, 2012) packages. OTUs classified as chloroplast and mitochondria were filtered out. The alpha diversity was estimated using the Shannon index and the Chao1 index using an absolute abundance matrix. The relationship between bacterial community structures from the two halophytes were evaluated using a principal coordinate analysis (PCoA) and complete linkage clustering (CLC) based on weighted UniFrac distances from the relative abundance matrix, and were statistically confirmed using PERMANOVA.

## Results

### General Characteristics of the Amplicons and Sequencing Data

In this study, we obtained 14,666,766 raw reads from the Miseq sequencing analysis of 24 samples, ranging from 1,138,891 to 334,380 reads per sample. After read-quality filtering, a total of 6,792,261 quality-filtered reads were obtained, ranging from 535,377 to 142,027 reads per sample with an average length of 416–419 bp. A total of 64,665 OTUs were extracted, ranging from 1,074 to 5,040 reads per sample (Supplementary Table S1).

**FIGURE 1:**
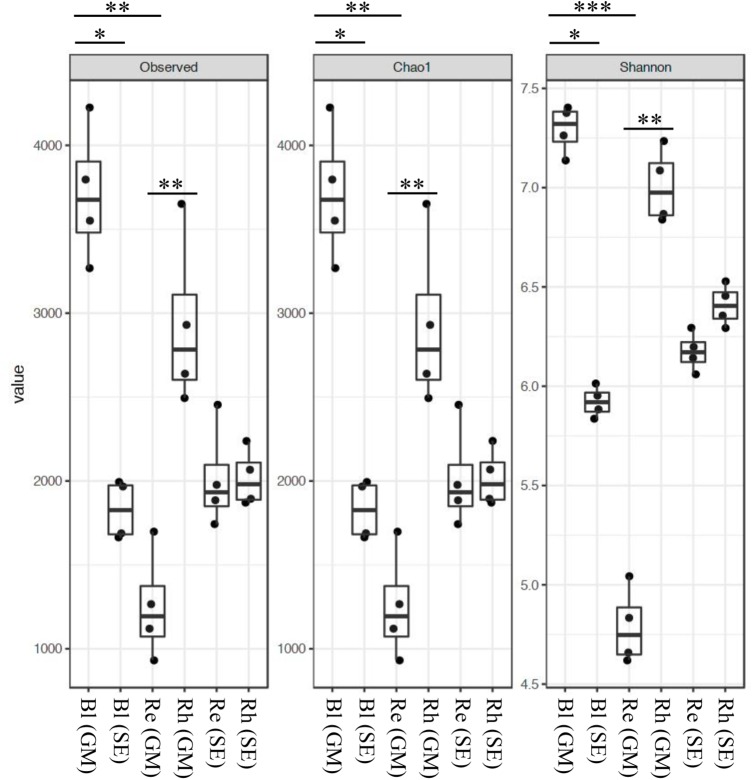
Alpha-diversity indices for the 16S rRNA gene sequences. Box plots of the observed OTUs, Chao1, and Shannon indices in bulk control soil (Bl), root endosphere (Re), and rhizosphere (Rh) samples from both *G*. *maritima* (GM) and *S*. *europaea* (SE). Whiskers represent the minimum and maximum values. All other points are contained within the box, and the bar represents the median. A Holm-adjusted *P*-value was calculated from Dunn’s test of multiple comparisons using rank sums. Asterisks indicate statistically significant differences between pairs of values (^∗^*P* < 0.05, ^∗∗^*P* < 0.01, ^∗∗∗^*P* < 0.001).

The rarefaction curves for the OTUs obtained from each sample are shown in Supplementary Figure S2. The highest richness was in the *G*. *maritima* Bl control soil sample, exhibiting significantly higher OTU (*P* = 0.0011), Chao1 (*P* = 0.0011), and Shannon (*P* = 0.0006) indices compared with the *G*. *maritima* Re samples. Furthermore, a comparison of alpha diversity metrics revealed a disparity in the OTU, Chao, and Shannon indices in the Rh samples from *G*. *maritima* compared with the Re samples (*P* = 0.0096, 0.0096, and 0.0081, respectively) (Figure 1 and Table 1). In contrast, the *S*. *europaea* Bl, Rh, and Re samples had similar numbers of OTUs, and both the Shannon and Chao1 indices exhibited a similar pattern to that of the OTUs (Figure 1 and Table 1).

**Table 1:** Summary of the richness and diversity indices of the three samples from both *G*. *maritima* and *S*. *europaea*.

Sample name	Sample origin	Plant species	OTU observed	Chao1	Shannon
Bl (GM)	Bulk control soil	*G. maritima*	3710 ± 405	3710 ± 405	7.29 ± 0.12
Rh (GM)	Rhizosphere	*G. maritima*	2929 ± 515	2928 ± 515	7.01 ± 0.19
Re (GM)	Root endosphere	*G. maritima*	1254 ± 326	1253 ± 326	4.79 ± 0.19
Bl (SE)	Bulk control soil	*S. europaea*	1828 ± 176	1828 ± 176	5.92 ± 0.08
Rh (SE)	Rhizosphere	*S. europaea*	2018 ± 171	2018 ± 171	6.41 ± 0.1
Re (SE)	Root endosphere	*S. europaea*	2015 ± 308	2015 ± 308	6.17 ± 0.1


*Values are the means of four replicates ± SD. Re, root endosphere; Rh, rhizosphere; Bl, bulk control soil; GM, *G*. *maritima*; SE, *S*. *europaea*.*

**FIGURE 2:**
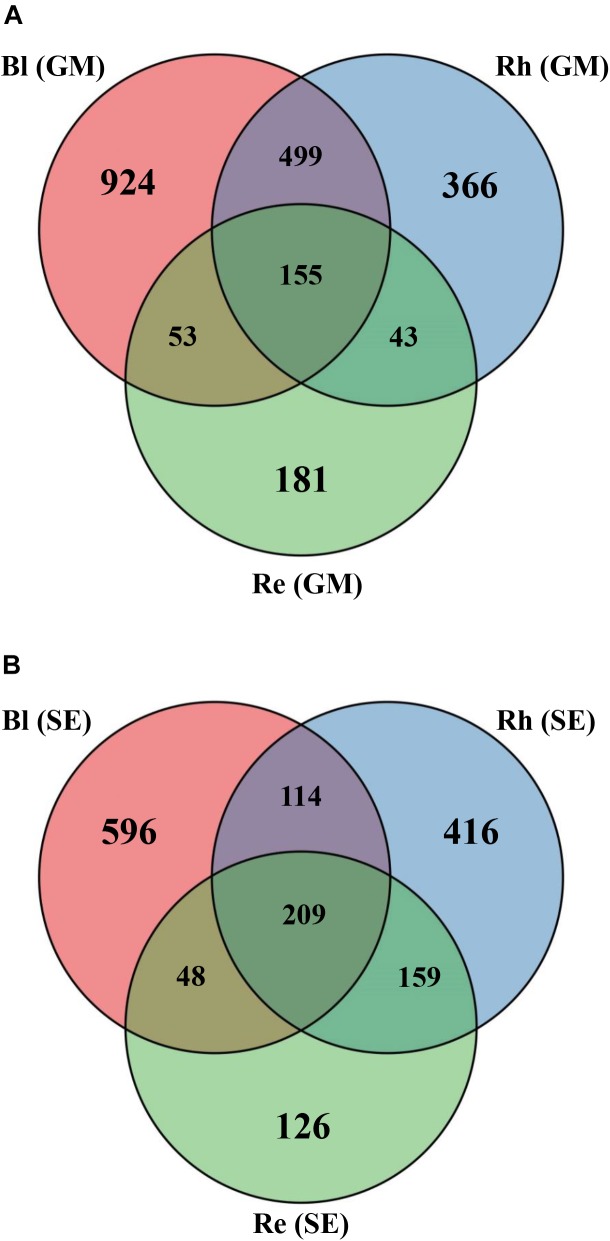
Venn diagrams showing the overlap of the OTU calculated separately for the **(A)** *G*. *maritima* and **(B)** *S*. *europaea* microbial communities. Re, root endosphere; Rh, rhizosphere; Bl, bulk control soil; GM, *G*. *maritima*; SE, *S*. *europaea*.

Based on a Venn diagram analysis, 155 OTUs were found to be common to all *G*. *maritima* samples. There were 924, 366, and 181 OTUs that were exclusive to the Bl, Rh, and Re *G*. *maritima* samples, respectively (Figure 2A). Similar results were also obtained in *S*. *europaea*. All samples shared 209 OTUs with 596, 416, and 126 OTUs being unique in the Bl, Rh, and Re samples, respectively (Figure 2B).

### Microbial Taxonomic Analysis at the Phylum and Class Levels

**FIGURE 3:**
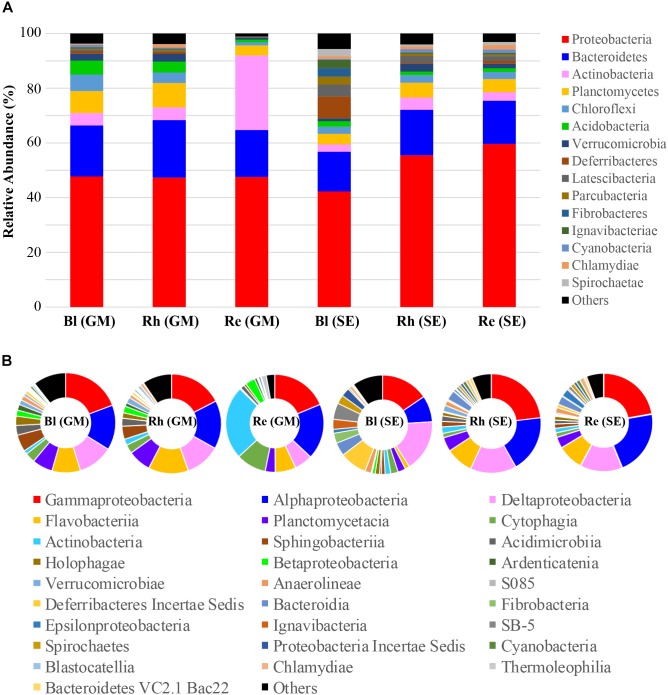
Average relative abundances of bacteria at **(A)** the phylum level and **(B)** the class level in the different samples. Re, root endosphere; Rh, rhizosphere; Bl, bulk control soil; GM, *G*. *maritima*; SE, *S*. *europaea*.

Classification of the high-quality sequences also demonstrated differences in the bacterial communities among the different samples at the phylum level. A total of fifty-seven phyla were identified in all samples. The relative abundance of the top 15 phyla (>1% of relative abundance in at least one sample) are shown in Figure 3A and Supplementary Table S2. *Proteobacteria* and *Bacteroidetes* were the dominant phyla (>10% relative abundance) across all samples, accounting for 42.2–59.6% and 14.5–20.9% of the total high-quality sequences, respectively. *Actinobacteria*, *Planctomycetes*, and *Chloroflexi* were the sub-dominant phyla (>1% relative abundance) in all samples, accounting for 2.7–27.2, 3.7–8.9, and 1.2–6.0 of the total high-quality sequences, respectively. Interestingly, the abundance of *Actinobacteria* in the *G*. *maritima* Re sample was extremely high, compared with that in the other samples. *Acidobacteria*, *Verrucomicrobia*, *Deferribacteres*, *Latescibacteria*, *Parcubacteria*, *Fibrobacteres*, *Ignavibacteriae*, *Cyanobacteria*, *Chlamydiae*, and *Spirochaetae* were present at > 1% relative abundance in at least one sample. The other 42 phyla had much lower abundances (less than 1% of the high-quality sequences). These phyla, therefore, were defined as rare phyla, and are referred to as “others” in Figure 3A.

A total of 151 bacterial classes were identified across all samples (Supplementary Table S3). There were 28 classes with a relative abundance of higher than 1% in at least one sample (Figure 3B and Supplementary Table S3). The other 123 classes with <1% abundance of the high-quality sequences, and are referred to as “others” in Figure 3B. Among these 28 classes, the more dominant classes (>5% relative abundance) in all samples were *Gammaproteobacteria*, *Alphaproteobacteria*, and *Deltaproteobacteria*, accounting for 14.0–21.9%, 7.8–20.7%, and 6.0–15.4% of the total high-quality sequences, respectively. The sub-dominant classes were *Flavobacteria*, *Planctomycetacia*, *Cytophagia*, and *Actinobacteria*, which had relative abundances of higher than 1% in all samples, especially the *G*. *maritima* Re sample, which possessed the highest abundance of *Cytophagia* and *Actinobacteria*, accounting for 9.9 and 24.3% of the total high-quality sequences, respectively. *Blastocatellia*, *Chlamydiae*, and *Thermoleophilia* had >1% abundance of high-quality sequences in the Rh and/or Re samples from *G*. *maritima*. Among the *S*. *europaea* Bl, Rh, and Re samples, the abundance of *Verrucomicrobia*, *Cyanobacteria*, *Chlamydiae*, and *Bacteroidetes* VC2.1 Bac22 were higher in the Rh and/or Re samples than in the Bl sample.

### Comparison of Bacterial Community Structure at the Family and Genus Levels

**FIGURE 4:**
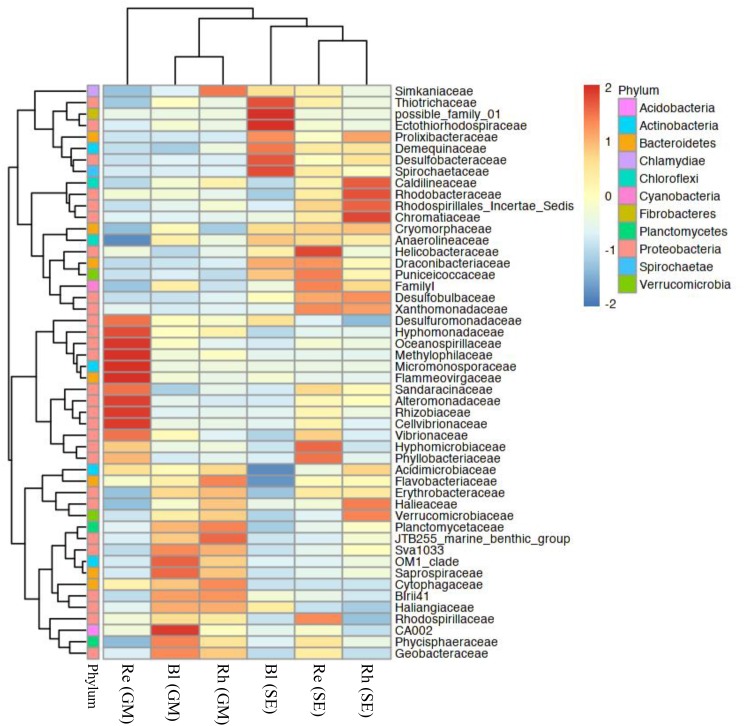
Heatmap of bacterial distribution of the top 50 abundant families in all sample types. The dendrogram shows complete-linkage agglomerative clustering based on a Euclidean distance. The heatmap color (blue to red) represents the row *z*-score of the mean relative abundance from low to high. Re, root endosphere; Rh, rhizosphere; Bl, bulk control soil; GM, *G*. *maritima*; SE, *S*. *europaea*.

Based on a heatmap analysis, the relative abundance of the top 50 classified families and genera clearly revealed that there were significant different bacterial community structures among the sample types. Figure 4 shows a clustering of the top 50 classified families. These classified families belonged to 11 phyla as shown in Supplementary Table S4. The distributions of the families differed greatly across the different samples. *Hyphomonadaceae*, *Oceanospirillaceae*, *Methylophilaceae*, *Micromonosporaceae*, *Flammeovirgaceae*, *Alteromonadaceae*, *Rhizobiaceae*, and *Cellvibrionaceae* were significantly abundant in the *G*. *maritima* Re samples, whereas only one family, *Helicobacteraceae*, was more abundant in the *S*. *europaea* Re samples. No family was significantly enriched in the *G*. *maritima* Rh samples. In contrast, *Caldilineaceae*, *Rhodobacteraceae*, and *Chromatiaceae* were more highly abundant in the Rh samples from *S*. *europaea*. CA002 was predominantly distributed in the *G*. *maritima* Bl samples. *Thiotrichaceae*, possible family 01, *Ectothiorhodospiraceae*, *Desulfobacteraceae*, and *Spirochaetaceae* were highly abundant in the *S*. *europaea* Bl samples.

**FIGURE 5:**
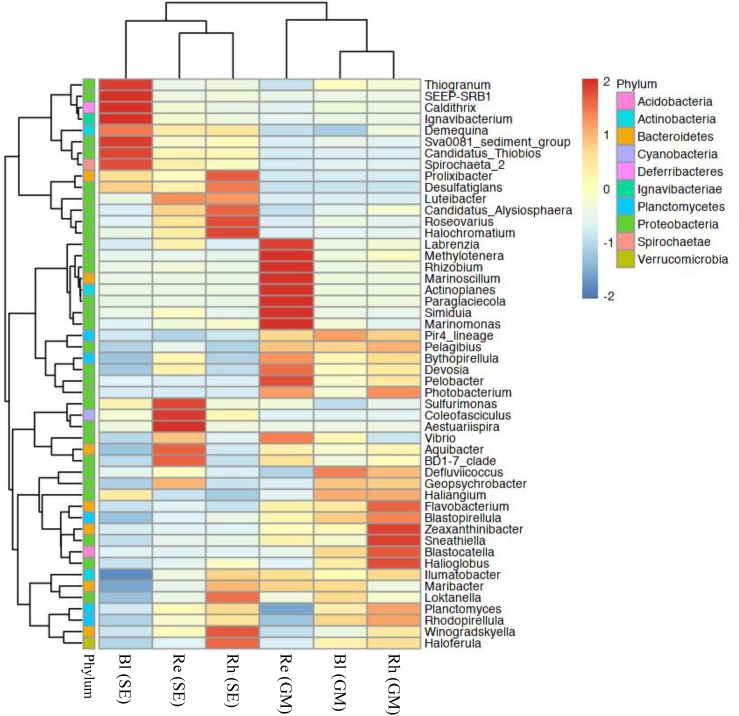
Heatmap of bacterial distribution of the top 50 abundant genera in all sample types. The dendrogram shows complete-linkage agglomerative clustering based on a Euclidean distance. The heatmap color (blue to red) represent the row *z*-score of the mean relative abundance from low to high. Re, root endosphere; Rh, rhizosphere; Bl, bulk control soil; GM, *G*. *maritima*; SE, *S*. *europaea*.

At the genus level, the top 50 classified bacterial genera belonged to 10 phyla as shown in Supplementary Table S5. From the heatmap shown in Figure 5, nine genera (*Labrenzia*, *Methylotenera*, *Rhizobium*, *Marinoscillum*, *Actinoplanes*, *Paraglaciecola*, *Simiduia*, *Marinomonas*, and *Pelobacter*) had were highly abundant in the *G*. *maritima* Re samples, whereas *Sulfurimonas*, *Coleofasciculus*, and *Aestuariispira* were significantly more abundant in the *S*. *europaea* Re samples. *Zeaxanthinibacter*, *Sneathiella*, *Blastocatella*, and *Halioglobus* were more abundant in the *G*. *maritima* Rh samples. In contrast, *Roseovarius* and *Halochromatium* were highly abundant in the *S*. *europaea* Rh samples. Although there was no significantly enriched genus in the *G*. *maritima* Bl samples, the following seven genera were dominant the *S*. *europaea* Bl sample: *Thiogranum*, SEEP-SRB1, *Caldithrix*, *Ignavibacterium*, Sva008 sediment group, *Candidatus Thiobios*, and *Spirochaeta* *2*.

### Comparative Analysis of Bacterial Communities in the Different Sample Groups

**FIGURE 6:**
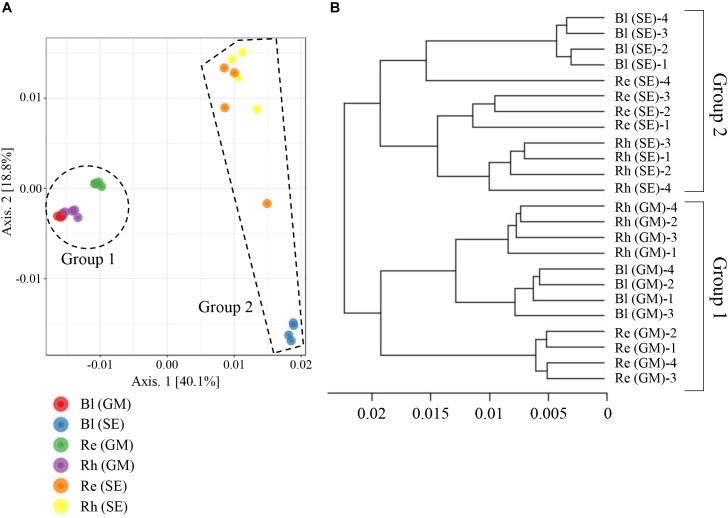
Principal coordinate analysis (PCoA) **(A)** and complete linkage clustering (CLC) **(B)** of the bacterial communities in different samples based on weighted UniFrac distances. Re, root endosphere; Rh, rhizosphere; Bl, bulk control soil; GM, *G*. *maritima*; SE, *S*. *europaea*.

A beta-diversity analysis based on PCoA (Figure 6A) and CLC (Figure 6B) was performed to compare the bacterial compositions among the different samples. All the samples were clustered into two groups by PCoA: the first containing the *G*. *maritima* Bl control soil, Rh, and Re samples (group 1 in Figure 6A) and the second containing the same *S*. *europaea* samples (group 2 in Figure 6A). This result shows that there is a strong separation based on plant species, which explained 40.1% (axis 1) of the variation. The quadruplicate Bl control soil, Rh, and Re samples obtained for each halophyte also clustered together, with the exception of the Re sample from *S*. *europaea*, explaining 18.8% (axis 2) of the variation (Figure 6A). Similar results were also obtained for the CLC tree. As shown in Figure 6B, the Re, Rh, and Bl samples from *G*. *maritima* (group 1) and the same samples from *S*. *europaea* (group 2) were also separated into two different clusters in the CLC tree. These results indicate that the microbiota in the Re, Rh, and Bl samples from *G*. *maritima* are largely different from those from *S*. *europaea*.

## Discussion

The halophytic plant-associated microbial community, and halotolerant PGPRs, play important roles in allowing hosts to adapt to a costal environment (Redman et al., 2002; Yuan et al., 2016; Etesami and Beattie, 2018). However, the diversity of halotolerant PGPRs present in saline containing soils and in halophyte-associated endophytic bacteria depends on the soil’s chemical and physical properties, as well as the plant species (Qin et al., 2016; Szymańska et al., 2016). To extend our knowledge about bacterial diversity related to halophytes, here we investigated the diversity and community structure of bacteria present in the rhizospheres and root endospheres of *G. maritima* and *S. europaea*. This study showed that the diversity of bacterial communities, including possible halotolerant PGPRs candidates in the rhizosphere and root endosphere, depends on the halophytic plant species and the sampling site.

The diversity and richness of bacteria in the rhizosphere from *G*. *maritima* were higher than those in the root endosphere. Previous reports have suggested that microbial density is generally higher in the rhizosphere than in the root, and that bacterial diversity and richness gradually decreases from the soil to the root-endosphere (Bulgarelli et al., 2012, 2015; Lundberg et al., 2012; Edwards et al., 2015; Hacquard et al., 2015). On the other hand, we found there were no significant differences in bacterial diversity and richness between the bulk control soil, rhizosphere, and the root endosphere for *S*. *europaea*. A similar result has also been reported for *Arabidopsis thaliana* (Lundberg et al., 2012) and *Bistorta vivipara* (Vik et al., 2013). Vik et al. (2013) concluded that there is a similarity in the micro-niches available for bacteria in plant roots and soil. However, a previous report has demonstrated that the bacterial diversity in the endosphere of *S*. *europaea* was lower than that in the rhizosphere of *S*. *europaea* (Shi et al., 2015). This difference indicates that the local environment has a complex effect on the bacterial community.

In all samples, the dominant bacterial phyla were *Proteobacteria*, *Bacteroidetes*, *Actinobacteria Planctomycetes*, and *Chloroflexi* (>1% of high-quality sequences). The bacterial species belonging to *Proteobacteria*, *Bacteroidetes*, and *Actinobacteria* could play important roles in the ecology of *G*. *maritima* and *S*. *europaea*, as these phyla are also dominant in other halophytes (Shi et al., 2015; Mukhtar et al., 2017; Tian and Zhang, 2017). Mukhtar et al. (2017) reported that *Planctomycetes* was also a dominant phylum in *Salsola stocksii*, similar to our results. Interestingly, the root endosphere of *G*. *maritima* has been shown to possess high levels of bacterial species from *Actinobacteria*. *Actinobacteria* have also been shown to be enriched in metabolically active cells in the *Arabidopsis* root (Bulgarelli et al., 2012; Reinhold-Hurek et al., 2015). Bulgarelli et al. (2013) suggested that the wide range of antimicrobial compounds secreted by members of *Actinobacteria* could play an important role, in that such compounds could indirectly protect sugar beet against soil-borne fungal pathogens. Therefore, it is possible that some members of *Actinobacteria* present in the root endosphere of *G*. *maritima* could also secrete antimicrobial compounds that could protect the host plant from fungal pathogens. Similar to our results, *Chloroflexi* was found in high abundance in mangrove sediments at a depth of 10 cm (Mendes and Tsai, 2014). Bacterial species in the phylum *Chloroflexi*, therefore, could play important roles in the decomposition of organic compounds in the coastal environment, as has been reported previously (Yamada et al., 2005; Mendes and Tsai, 2014). The dominant bacterial classes were *Gammaproteobacteria*, *Alphaproteobacteria*, *Deltaproteobacteria*, *Flavobacteria*, *Planctomycetacia*, *Cytophagia*, and *Actinobacteria* (>1% of high-quality sequences) in all samples. Unlike our study, Yuan et al. (2016) reported that *Deltaproteobacteria*, *Alphaproteobacteria*, *Bacteroidetes*, and *Verrucomicrobia* were the dominant classes in the halophyte *S*. *salsa* using two primer sets that amplified the V3-V4 and V5-V9 regions. The choice of primers might have a significant effect on the observed differences in bacterial communities, as has been described previously (Klindworth et al., 2013; Thijs et al., 2017), as we only selected primers spanning the V3-V4 region of the bacterial 16S rRNA gene in this study. In addition, this difference might be a reflection of differences in plant species, sampling methods, and/or sampling area. In this regard, in a pristine mangrove, the bacterial composition has been shown to change dramatically according to the depth of the sediment samples (Mendes and Tsai, 2014).

The beta-diversity analyses showed that bacterial communities varied across the different plant species. These results were also supported by heatmap analyses at the family and genus levels (Figures 4, 5). As demonstrated by a PCoA, plant species explained 40.1% of the variation, whereas sample type explained 18.8% of the variation (Figure 6A). Similar to this, Dong et al. (2018) reported that the community structures of the root microbiome were significantly different among four different sugarcane species. Therefore, the data from this study and previous investigations indicate that plant species have a significant influence on bacterial communities.

For the top 50 genera, we have summarized the possible functions of the abundant bacterial genera in the rhizosphere and root endosphere from the two halophytes, as shown in Tables 2A,B. In the root endosphere and rhizosphere of *G*. *maritima*, many species of *Actinoplanes*, *Marinomonas*, and *Rhizobium* have been reported to have beneficial effects for host plants, such as the production of indole-3-acetic acid (IAA), indole-3-pyruvic acid (IPYA), and gibberellic acid (GA_3_), antifungal activity, N_2_-fixation, phosphate solubilization, siderophore production, and 1-aminocyclopropane-1-carboxylate (ACC) deaminase activity (Table 2A). Furthermore, several species of *Rhizobium* have been isolated as halotolerant PGPRs from three halophytes, *Psoralea corylifolia*, *Salicornia brachiate*, and *Salicornia bigelovii* (Etesami and Beattie, 2018). These three genera, therefore, could be possible candidates as halotolerant PGPRs for *G*. *maritima*. In addition, *Pelobacter* was also a dominant genus in the root endosphere from *G*. *maritima*. *Pelobacter propionicus* can use C2 compounds such as lactate, pyruvate, 2,3-butanediol, acetoin, and ethanol for growth under strictly anaerobic conditions, and can thereby induce propionate formation (Li et al., 2010). Methyl 3-(4-hydroxyphenyl) propionate functions as a modulator of the architecture of the root system (Liu et al., 2016). The propionate induced by *Pelobacter*, therefore, might regulate root formation in *G*. *maritima*. On the other hand, the *Coleofasciculus* (*Microcoleus* sp.) genus, which is related to N_2_-fixation, suggests a possible function in the root endosphere of *S*. *europaea* (Table 2B). Toledo et al. (1995) reported that *Coleofasciculus* can form a biofilm on the root of the black mangrove, in which the cyanobacterial filaments are embedded and may be capable of N_2_-fixation, although ^15^N assimilation tests have not yet been performed to confirm this. In addition, the sulfur-oxidizing genera, *Sulfurimonas* and *Halochromatium*, were significantly abundant in the root endosphere and rhizosphere, respectively, in *S*. *europaea* (Table 2B). According to a previous report, *Sulfurimonas* might be related to host detoxification by oxidizing sulfide and producing sulfate as an end product, suggesting that the accumulation of these bacteria around the rhizosphere might be critical for the host tolerance of coastal environments (Fahimipour et al., 2017). Hasler-Sheetal and Holmer (2015) also suggested that the oxidation of sulfide and the production of non-toxic S^0^ in the aerenchymous tissue of seagrass is involved in the detoxification mechanisms of the host plants. Thus, *Coleofasciculus* and *Sulfurimonas* could play important roles as halotolerant PGPRs in *S*. *europaea*. The possible function of any of the other bacteria in their host plants, unfortunately, is still unknown (Tables 2A,B).

**Table 2A:** Possible function of the abundant bacterial genera in the root endosphere and rhizosphere in *G*. *maritima.*

Genus	Sample type	Possible function	Reference
*Actinoplanes*	Re	IAA, IPYA and GA_3_ productions, antifungal activity.	El-Tarabily et al., 2009
*Marinoscillum*	Re	Unknown. One of dominant genera in rhizosphere of *S. salsa.*	Yuan et al., 2016
*Pelobacter*	Re	Propionate formation as a modulator of the root system architecture.	Li et al., 2010; Liu et al., 2016
*Marinomonas*	Re	N_2_-fixation, phosphate solubilization, IAA production, siderophore production, ACC deaminase activity.	Mesa et al., 2015
*Methylotenera*	Re	Unknown.	—
*Paraglaciecola*	Re	Unknown.	—
*Rhizobium*	Re	N_2_-fixation, phosphate solubilization, IAA production, siderophore production, ACC deaminase activity.	Jha et al., 2012; Sorty et al., 2016
*Simiduia*	Re	Degradation of a variety of refractory polysaccharides. There are no reports about beneficial function to host plants.	Lin et al., 2013
*Labrenzia*	Re	Unknown. Isolated from root endosphere of halophytes.	Bibi et al., 2014; Fidalgo et al., 2016
*Zeaxanthinibacter*	Rh	Zeaxanthin-producing marine bacterium. There are no reports about beneficial function to host plants.	Asker et al., 2007
*Sneathiella*	Rh	Unknown.	—
*Blastocatella*	Rh	Unknown. One of abundant genera present in the rice rhizosphere.	Osman et al., 2017
*Halioglobus*	Rh	Unknown.	—


*Re, root endosphere; Rh, rhizosphere.*

**Table 2B:** Possible function of the abundant bacterial genera in the root endosphere and rhizosphere in *S*. *europaea.*

Genus	Sample type	Possible function	Reference
*Coleofasciculus*	Re	N_2_-fixation.	Toledo et al., 1995
*Aestuariispira*	Re	Unknown.	—
*Sulfurimonas*	Re	Host detoxification by the oxidation of sulfide.	Hasler-Sheetal and Holmer, 2015; Fahimipour et al., 2017
*Halochromatium*	Rh	Sulfur-oxidizing activity. There are no reports about beneficial function to host plants.	Montoya et al., 2011
*Roseovarius*	Rh	Unknown.	—


*Re, root endosphere; Rh, rhizosphere.*

Taken together, our results suggest that there are apparent differences in the bacterial communities, and in the different varieties of beneficial bacteria, found between *G. maritima* and *S. europaea*. Further research is required to clarify whether there are specific interactions between the bacteria enriched in the two halophytes and their host.

## Conclusion

This report is the first to clarify the bacterial diversity and community structure of a halophyte, *G*. *maritima*, using next-generation sequencing technology and to compare the diversity and composition with those of *S*. *europaea*. The bacterial community structures varied among the three sample types, namely the root endosphere, the rhizosphere, and the bulk control soil in each halophyte. The more dominant bacteria phyla associated with both *G*. *maritima* and *S*. *europaea* were *Proteobacteria* and *Bacteroidetes*. Beneficial bacterial genera were enriched in the root endosphere and the rhizosphere in both halophytes. *Actinoplanes*, *Marinomonas*, and *Rhizobium* were found to be significantly abundant in *G*. *maritima*, whereas *Sulfurimonas* and *Coleofasciculus* were more highly abundant in *S*. *europaea.* Our results indicate that there are clear differences in bacterial diversity and community structure between *G*. *maritima* and *S*. *europaea*. These results provide a new insight into the complex bacterial community structures of halophytes. In the future, we plan to investigate the functional roles of these potential beneficial bacteria in the interaction between plants and microbes in coastal areas.

## Author Contributions

KY contributed to the conception of the study, performed the experiments and data analyses, and wrote the manuscript. YS performed data analyses. TI performed the experiments and data analyses. HkS collected the samples. KT, MU, NT, SO, HmS, and ST contributed to a fruitful discussion. All authors contributed to manuscript revision and have read and approved the submitted version.

## Conflict of Interest Statement

The authors declare that the research was conducted in the absence of any commercial or financial relationships that could be construed as a potential conflict of interest.

## Footnotes

1 https://qiime2.org/

2 http://www.arb-silva.de
