# Supplementary material for: Bacterial Diversity Associated With the Rhizosphere and Endosphere of Two Halophytes: Glaux maritima and Salicornia europaea
Source: Front Microbiol. 2018 Nov 28;9:2878. doi: 10.3389/fmicb.2018.02878 (PMC6282094; doi:10.3389/fmicb.2018.02878)

FIGURE S1 | The sampling site was located at 'Lake Notoro' in the eastern part of Hokkaido, Japan **(A)** and is shown by the magnified image of the selected area **(B)** using Google Earth software. *Glaux maritima* **(C and E)** and *Salicornia europaea* **(D and F)** grow in two different tidal areas at Lake Notoro. The two halophytes were harvested and the root systems sampled as described in the Materials and Methods.

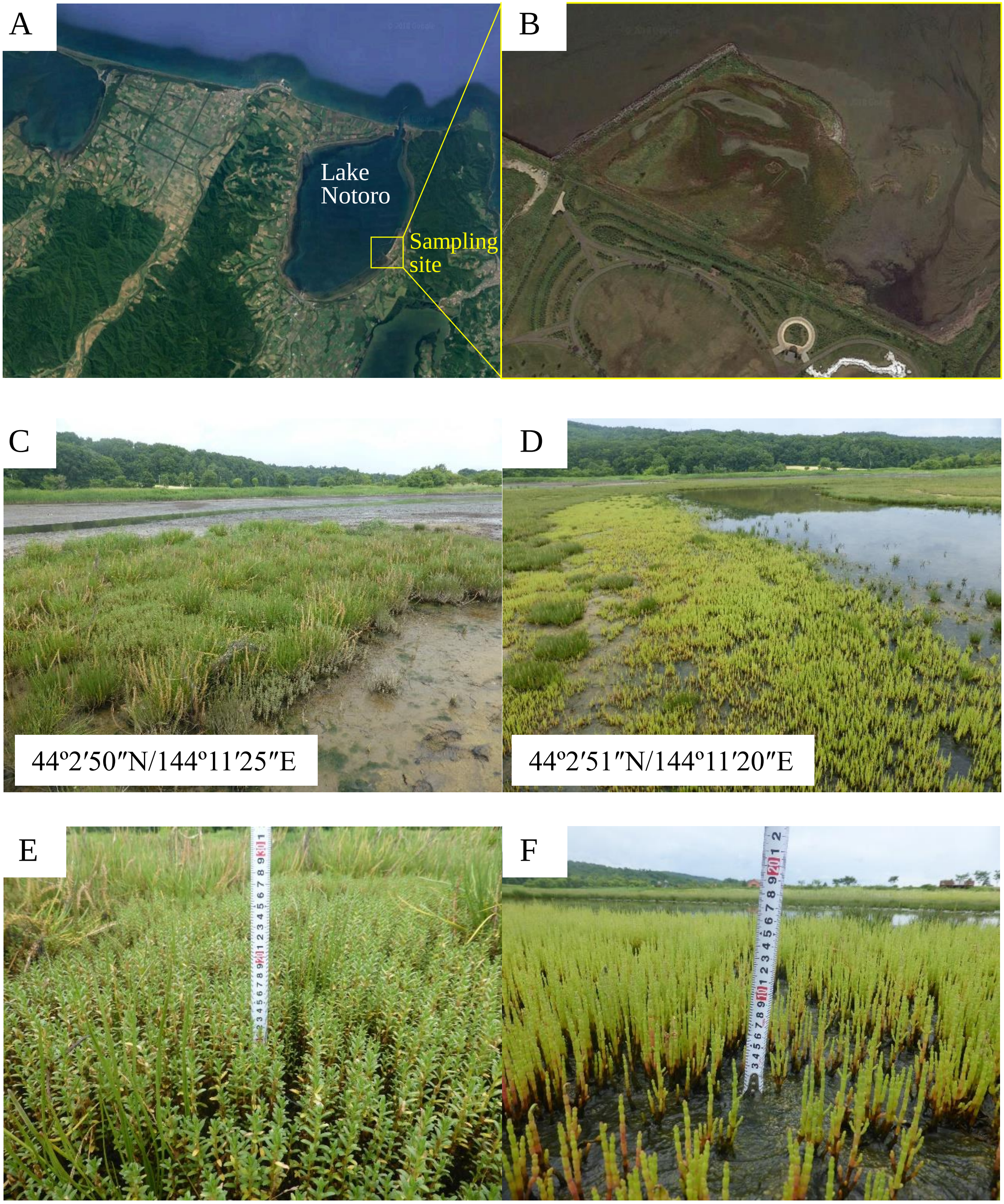

Supplement: Supplementary file 6 [file Data_Sheet_1.PDF]
