# Supplementary material for: Bacterial Diversity Associated With the Rhizosphere and Endosphere of Two Halophytes: Glaux maritima and Salicornia europaea
Source: Front Microbiol. 2018 Nov 28;9:2878. doi: 10.3389/fmicb.2018.02878 (PMC6282094; doi:10.3389/fmicb.2018.02878)

FIGURE S2 | Rarefaction curves based on the sequences of the V3–V4 region of the 16S rRNA gene from each sample. The error bars represent the standard error of four replicates. Re, root endosphere; Rh, rhizosphere; Bl, bulk control soil; GM, *G. maritima*; SE, *S. europaea*.

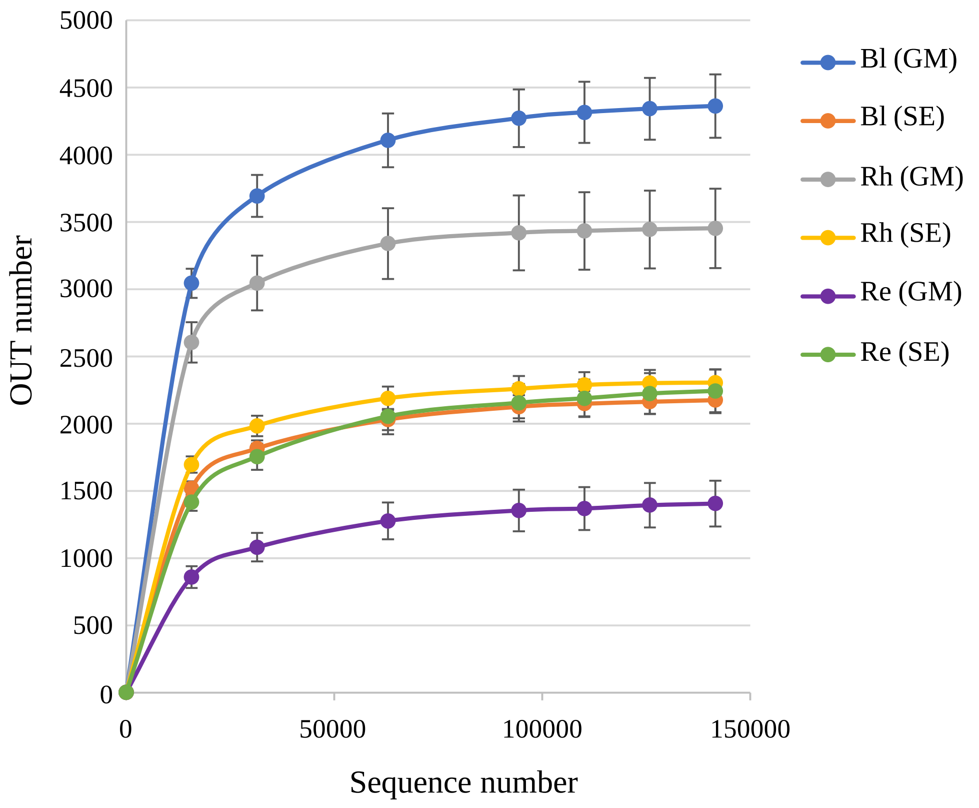

Supplement: Supplementary file 7 [file Data_Sheet_2.PDF]
